# Gold nanoparticles synthesis mediated by fungus isolated from aerobic granular sludge: Process and mechanisms

**DOI:** 10.1016/j.heliyon.2024.e28281

**Published:** 2024-03-16

**Authors:** Xin Zhao, Ning Hou, Chunli Wan, Lei Zhang, Xiang Liu

**Affiliations:** a College of Resources and Environment, Northeast Agricultural University, Harbin, Heilongjiang, 150030, China; b Department of Environmental Science and Engineering, Fudan University, Shanghai, 200438, China; c School of Civil and Environmental Engineering, Queensland University of Technology, Brisbane, 4001, Australia

**Keywords:** Fungus, Gold nanoparticles, Aerobic granular sludge, Reducing polysaccharides

## Abstract

Due to the low toxicity, biocompatibility and eco-friendliness, microorganisms have received a lot of attention for gold nanoparticles (AuNPs) synthesis. This work isolated a fungal strain capable of efficiently generating AuNPs from aerobic granular sludge, named XY3. Comparison of 18S rDNA sequence results showed that fungus XY3 belongs to *Candida rugopelliculosa*. AuNPs were synthesized by initiating an Au^3+^-induced stress response that prompted the reduction of Au^3+^ to Au^0^ by the fungus XY3. It is worth noting that the addition of nutritional substrates weakens the stress response induced by Au^3+^, resulting in a decrease in the yield of AuNPs. As evidenced by nystatin inhibition studies, the synthesis of AuNPs is based on biochemical reactions rather than purely physical changes. The XRD results suggested that XY3-secreted biomolecules were involved in the reduction of Au^3+^ and AuNPs synthesis. The results of the three variation patterns of reducing power, biomolecules, and AuNPs absorbance revealed that Au^3+^ reduction was mostly dependent on the reducing polysaccharides. In addition, extracellular proteins were shown to be involved in the synthesis of AuNPs, which is responsible for the uniform distribution of AuNPs. This work provided a wide and cost-effective seed source for AuNPs synthesis, and also offered a resourceful solution for residual sludge treatment of fungal type aerobic granular sludge.

## Highlights

• A green AuNPs synthesized fungus was firstly isolated from aerobic granular sludge.
• Fungus XY3 had 99% of similarity with *Candida rugopelliculosa.*
• AuNPs synthesis by fungus XY3 was dependent to biological metabolization.
• Reducing polysaccharides on surface of fungus XY3 was responsible for Au^3+^ reduction.
• Proteins on surface of fungus XY3 was responsible for uniform dispersion of AuNPs.

## 1 Introduction

Nanoparticles generally refer to materials with a particle size range of 1–100 nm, which usually have special physical, chemical, and biological properties and have been widely used in electronic devices, medical, environmental and biotechnological applications, etc [1]. Gold is a crucial precious metal, which is scarce and costly in nature. Au in nano form have outstanding optoelectronic capabilities, biocompatibility, and good specific surface area, which can remain stable in complex environmental systems. As a result, it is indispensable in a wide range of industries, including analysis and detection, medical health, electronic technology, reaction catalysis, and so on [[1], [2], [3], [4], [5], [6]].

At present, gold nanoparticles (AuNPs) synthesis mainly relied on physical, chemical, and biological methods. The physical approach disperses the gold colloid by radiation and ultrasound [7,8]. The finer the particle size required, the higher the energy input. This method produces AuNPs with high purity and uniform distribution, but the reaction conditions are more demanding, and the input cost is higher. Chemical synthesis mainly includes redox and electrochemical reduction. Sodium citrate, sodium borohydride, and ascorbic acid are the common chemicals reducing Au(III) to elemental Au monomers in the zero valence state [3,9]. In electrochemical preparation, [AuBr_4_]^-^ or [AuBr_2_]^-^ was covered by cetyltrimethylammonium bromide (CATB) in the electrolyte, after which the Au(III) salt or Au(I) salt gains electrons to be reduced to Au^0^ by the catalytic action of the gold seeds [10]. Although chemical synthesis is very efficient, it is frequently required to add a stabilizer to the system to prevent AuNPs aggregation. Common stabilizers include amines, carboxylic acids, phosphorus-containing, and sulfur-containing compounds [11]. Seed-mediated growth, surface modification, and entrapment methods have further been established, to alter and adjust the distribution form and existing state of gold nanoparticles by employing various types of stabilizers [12,13]. Therefore, chemical synthesis is convenient for controlling the size and morphology of gold nanoparticles, and the preparation is tailored to specific applications. However, chemical methods are more complicated, requiring precise reagent dosing and condition monitoring, as well as the subsequent separation and purification of AuNPs. As a result, the toxic reagents and high costs associated with physical and chemical synthesis have become a major source of obstacles [14]. In addition, the extraction of nanoparticles from plant extracts is also a hot research topic [15,16]. Due to the large amount of phytochemicals contained in the extracts, the reaction rate and pathway may not be easy to control, thus restricting the development of this technology [17]. Biological techniques have gained popularity since microorganisms can not only metabolize Au(III) but also stabilize AuNPs due to their unique structure. Microorganisms have been widely studied and applied as a widely available, inexpensive, and environmentally adaptable reducing agent. Currently, bacteria, actinomycetes and fungi have been shown to be used for the efficient synthesis of AuNPs [18]. Due to the low toxicity, biocompatibility, and eco-friendliness of microorganisms, the biological pathway of synthesizing metal nanoparticles by microorganisms has received a lot of attention. However, there is still a need for screening efficient microorganisms, exploring the synthesis mechanism, and optimizing the synthesis conditions [19].

Microbial synthesis of AuNPs is primarily a stress response to Au(III) toxicity, with surface binding, intracellular aggregation, and extracellular precipitation as the key reaction mechanisms [20,21]. Microbial metabolic activities are required for intracellular aggregation and extracellular precipitation. Metal ion entry and exit are primarily controlled by energy-intensive transport processes, but when the intracellular concentration is too high, the metal must be stabilized intracellularly or extracellularly by secreting organic substances in the form of complexation, precipitation, reduction, and other processes [22]. Extracellular stabilization of metals is also known as a microbial mineralization process. Biomolecules (e.g. proteins, peptides, polysaccharides, and organic acids) have been proven to stabilize reduction, allowing Au to remain stable at the nanoscale [23,24]. Compared to bacteria, fungi have several advantages in AuNPs biosynthesis [[25], [26], [27], [28], [29]], including 1) fungi have higher metal ion binding capacity and higher tolerance concentration; 2) fungi can exude vast volumes of extracellular organic materials, which can be employed as reducing media and capping agents; 3) fungal mycelium is more stable under agitation and high flow pressure; 4) smaller fungal-mediated synthesis of AuNPs can be achieved, resulting in greater quantum yields and biocompatibility. There are few investigations on fungal-mediated AuNPs production, and it has been discovered that different genera of the same species of fungi have varied adsorption and reduction capacities [30]. Therefore, it is of great practical value to continue screening for fungi with the ability to mediate AuNPs formation and explore their mediating mechanisms.

Based on this, this work used fungal aerobic granular sludge used in wastewater treatment as the seed source, screened the fungus from fungal aerobic granular sludge that could mediate rapid AuNPs production, and investigated the strain's mediation mechanism. This work could offer a diverse set of cost-efficient and effective seed sources for AuNPs production, as well as practical options on for residual sludge treatment of fungal-type aerobic granular sludge.

## 2 Materials and methods

### 2.1 Screening and identification of fungi

The fungal aerobic granular sludge (AGS) was adopted to isolate the target fungi. The fungal aerobic granular sludge was cultivated referring to the culture method of the previous study [31]. The sequencing batch reactor was a column with the total volume of 3.5 L (working volume of 2.0 L). The inner diameter and height were 5 cm and 180 cm, respectively. The volumetric exchange rate was 70%.The influent volume in each cycle was 1.6 L. The influent components include 0.2 g/L of NH_4_Cl, 0.2 g/L of KH_2_PO_4_, 0.03 g/L of CaCl_2_, 0.025 g/L of MgSO_4_·7H_2_O, 0.02 g/L of FeSO_4_·5H_2_O, 0.013 g/L of NaHCO_3_, 0.04 g/L of peptone. The aeration volume was 5 L/min. Six cycles were included in each day. Each cycle was 4 h, with 3 min influent, 227 min of aeration and settling, 5 min of discharge, and 5 min of idle phase. The settling time is modified during operation following the particle cultivation process, and the aeration time increases as the settling time lowers. Glucose was used as carbon source. The COD was adjusted as following program: 1000 mg/L for 1–3 d, 3000 mg/L for 4–6 d, 1000 mg/L for 7–9 d, 3000 mg/L for 10–13 d. Fungal AGS could be obtained on Day 13, with an average diameter of 3–8 mm (Fig. S1(a)). Used 3 mm AGS as the inoculum source, target fungi were isolated used Sabourand's medium, which contained 40 g/L glucose, 10 g/L peptone, and 20 g/L agar in solid medium. A small amount of fungal organism was picked and added to 1 mM HAuCl_4_·4H_2_O solution. The solution was cultivated at 35 °C, 120 rpm of rotation speed, for 12 h. At the end of the incubation, the strain that could significantly convert HAuCl_4_·4H_2_O into AuNPs (dark red solution and showing an absorption at 540 nm) was chosen and named XY3.

Population analysis was taken by using 18S rRNA gene-based clone libraries. Genomic DNA was extracted using a fungal genome extraction kit (Sangon Biotech Co., Ltd, Shanghai, Chian), and 50 μL of genomic DNA solution was finally obtained. Universal primers (NS7 and NS8) were used to extract 18S rRNA [32,33], corresponding to the V8 section, the 50 μL PCR amplification system included: 5 μL of 10 × LA Taq buffer, 8 μL of 2.5 mM dNTP, 1.5 μL of each primer (20 μM/L), 2.5 U of La Taq, 1 ng sample. PCR program includes 94 °C 10 min, 30 cycles including 90 °C 1 min, 50 °C 1 min, and 72 °C 1 min 30 s, 72 °C 10 min. DNA and PCR products were confirmed by using 1% agarose. PCR amplification reagents were purchased from Takara Co., Ltd, Dalian, China. The primer sequences are listed as follow: NS7: 5′-ATAACAGGTCTGTGATGC-3′, NS8: 5′-CGCAGGTTCACCTACGGA-3'. The non-labeled PCR products were purified and recovered by using agarose electrophoresis. Recovered fragments were connected to pMD 18-T. *E Coli.* DH5α was used as host cells. Finally, the positive clones were randomly selected and sent to Shanghai Biotech for sequencing analysis. The sequences were compared with known sequences on NCBI.

### 2.2 AuNPs synthesis promoted by fungus XY3

#### 2.2.1 AuNP formation mediated by fungus XY3

Pure pseudo filamentous yeast was taken on a solid medium uninoculated it into 200 mL of liquid Sabourand's medium, and placed it in a shaker to cultivate (35 °C, 120 rpm). After incubating for 12 h, 100 mL of the fungus solution was taken for concentration determination, and the concentration was approximately 1540 ± 42 mg/L. Then, 5 mL of the mixed solution was transferred into two centrifuge tubes and centrifuged at a speed of 5000 rpm for 10 min. Referring to a related study [34], the precipitate was washed several times with 0.9% NaCl solution until complete removal of by-products, and then transferred to two 10 mL HACH colorimetric tubes. Four sets of tests were set up as follows: 1) the first set contained fungus precipitate and 5 mL deionized water; 2) the second set contained fungus precipitate and 5 mL Sabourand's medium; 3) the third set contained 5 mL deionized water; 4) the fourth set contained 5 mL Sabourand's medium. The above-mentioned four tests were added with HAuCl_4_·4H_2_O, with a final concentration of 1 mM. Cultivated under conditions of 35 °C and 120 rpm. The samples were taken at 4, 12, 24, 36, and 48 h. AuNPs were determined by UV–visible spectrum.

#### 2.2.2 Fungi-mediated AuNPs synthesis inhibited by nystatin

As described in section 2.2.1, re-cultured fungus XY3 medium (1713 ± 57 mg/L) were respectively loaded in three centrifuge tubes (5 mL for each), and washed with 0.9% NaCl solution by three times. The precipitate was divided into four sets of tests as follows: 1) the first set contained fungus precipitate; 2) the second set contained fungus precipitate and 5 mL nystatin (10 μg/L); 3) the third set contained nystatin pretreated fungus precipitate and 5 mL deionized water; 4) the fourth set contained 5 mL nystatin. The last three sets were added with HAuCl_4_·4H_2_O, with a final concentration of 1 mM. The mixture was cultured at 35 °C, 120 rpm. The samples were taken at 4, 12, 24, 36, and 48 h. AuNPs were determined by UV–visible spectrum. In the second group 20 μL of the mixture was taken and incubated in solid medium to verify the inhibitory effect of nystatin acid on fungus.

#### 2.2.3 Effects of XY3 surface structure on AuNPs synthesis

As described in section 2.2.1, re-cultured fungus XY3 medium (1713 ± 57 mg/L) were respectively loaded in three centrifuge tubes (5 mL for each), and washed with 0.9% NaCl solution for three times. Two of them were used for surface modification, including –COOH modification (the washed biomass was suspended in 100 mL of anhydrous methanol and the suspension was modified by adding 1% concentrated hydrochloric acid) [35] and –NH_2_ modification (the washed biomass was mixed and suspended with methyl iodide at a ratio of 1/20 and stirred for 4 h at room temperature (30 °C) for modification) [36]. Three sets of tests were taken as follows: 1) the first set contained un-modified bacterial precipitates and 5 mL deionized water; 2) the second set contained –COOH modified bacterial precipitates and 5 mL deionized water; 3) the third set contained –NH_2_ modified bacterial precipitates and 5 mL deionized water. Three sets were all added with HAuCl_4_·4H_2_O, with a final concentration of 1 mM. The mixture was cultured at 35 °C, 120 rpm. The samples were taken at 4, 12, 24, 36, and 48 h. AuNPs were determined by UV–visible spectrum.

### 2.3 Analytical methods

Fungus content is determined by the weight method. The oven temperature is 25 °C, while the retention time is 2 h. AuNPs in solution was scanned using UV–visible spectrometer (HACH, DR5000) with baseline normalization. The content of protein and polysaccharides in soluble extracellular polymers secreted by fungi was determined by the Lowry method [37] and phenol-sulfuric acid method [38]. Pure fungal cells were obtained after centrifugal washing of fungal liquid and modified fungal liquid. After that, a small amount of fungal cells were taken and dried at 45 °C for 1h and mixed with appropriate amount of KBr, and the surface taxa of fungal cells were analyzed by fourier transform infrared spectrometer (FTIR). The samples were pre-treated by high-speed centrifugation and freeze-drying, and then the structure of fungal cells and AuNPs were examined by scanning electron microscopy. The reducing power was determined by the slightly modified Oyaizu's method [39,40], which detects the reducing power by the amount of Fe^3+^ reduced to Fe^2+^ by the solution to be tested. The sample to be tested was taken and reacted by adding the required reagents according to the standard Oyaizu's method and finally the absorbance value was measured at 700 nm.

## 3 Results and discussion

### 3.1 Characterization of fungal XY3 and AuNPs synthesis

Comparison results of 18S rDNA sequence of fungus XY3 and NCBI data indicated 99% similarity to *Candida rugopelliculosa* (NG063475.1), which belongs to the *Pichia/Candida* clade under *Saccharomyceta* and includes extensive mycelium (Fig. S1(a-c)).

**Fig. 1:**
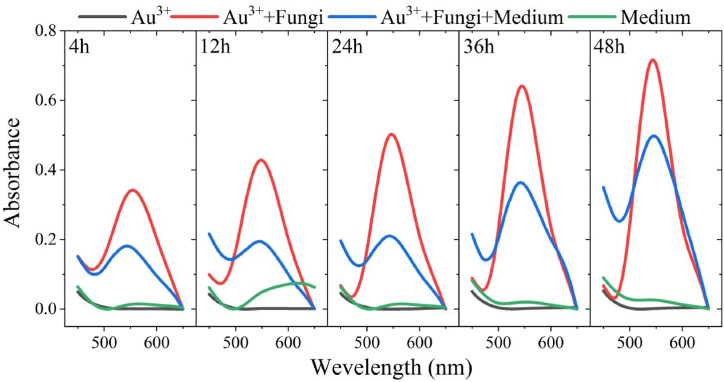
UV–Vis spectra at 4 h, 12 h, 24 h, 36 h and 48 h after addition of different components.

In the test group with the addition of fungus XY3, the reaction solution gradually changed from clear to burgundy with increasing incubation time (Fig. S2). The UV–visible spectrum showed that a clear absorption peak at 540–550 nm was observed (Fig. 1), which was in accordance with the basic characters of AuNPs [39,40]. Only the simultaneous presence of XY3 and Au^3+^ promoted AuNPs formation. The XY3 exhibited a strong ability to reduce Au^3+^ when stimulated with 1 mM HAuCl_4_·4H_2_O. The increasing absorbance of 0.341, 0.428, 0.502, 0.641, and 0.715 were respectively observed at 4 h, 12 h, 24 h, and 36 h, representing that the AuNPs amount was gradually increased. The coexistence of XY3, medium, and Au^3+^ also promoted AuNPs formation. The absorbance was gradually increased with time, with absorbances of 0.181, 0.195, 0.210, 0.363, and 0.497 at 4 h, 12 h, 24 h, 36 h, and 48 h, respectively. Whereas no AuNPs were obtained in any of the experiments without the addition of the fungus, implying that AuNPs production is dependent on the XY3. According to Kulkarni et al. [41], fungi's metal-reducing ability is related to microorganisms' stress response to metal ions, which was validated in the present study. After the addition of the medium, the fungus' ability to proliferate could lessen the stimulating effect of 1 mM HAuCl_4_·4H_2_O, and the nutritional substrate in the medium can also bind to Au^3+^, reducing the stimulating action and therefore resulting in a reduced AuNPs content. As a result, when converting Au^3+^ to AuNPs through stress-induced reaction by fungus XY3, the AuNP yield was higher when HAuCl_4_·4H_2_O was added alone compared to when both HAuCl_4_·4H_2_O and the growth medium were added.

### 3.2 Role of AuNPs and extracellular secretions

**Fig. 2:**
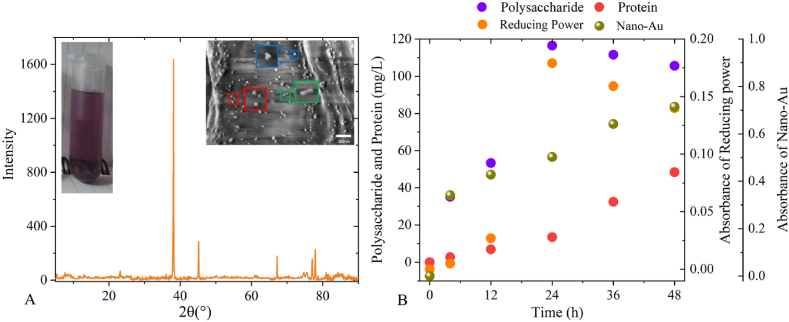
(A) Morphology and XRD patterns of AuNPs synthesized by the fungus XY3. (B) Determination of reducing power and extracellular biomolecule secretion.

Fig. 2 showed a more detailed study of the AuNPs production process when only fungus XY3 and Au^3+^ were present. The AuNPs generated were mostly suspended in liquid, with a small amount on the cell surface (Fig. 2A). The particle size ranged from 10 to 30 nm, which was comparable to the particle size range (8–40 nm) of *Fusarium oxysporum* [42], *Verticillium* sp. [43], and *Colletotrichum* sp. [44] promoting AuNPs formation reported in previous studies. Because the spherical shape of nanoparticles, which is the most frequent form of AuNPs, ensures that it is at the lowest free energy, it can sustain stable performance [45]. In addition, there are a few rods and triangles, which is comparable to the phenomenon described by previous study [46]. According to the mineralization process and mechanism of microorganisms [25], there should be bio-organic macromolecules involved in the AuNPs development. The diversity of macromolecular structures and functional groups leads to the diversity of nanoparticle shape [47]. XRD patterns revealed that XY3 mediated the AuNPs synthesis, with distinct diffraction peaks at 38°, 45°, and 67°, which are similar to the findings of He et al. [48]. These three typical diffraction peaks are consistent with polycrystalline gold with face-centered cubic unit cell features [49].

Fig. 2B shows the findings of the extracellular secretion analysis. With the prolongation of the reaction time, XY3 produced polysaccharides and proteins through endogenous metabolism to resist Au^3+^ stimulation, in absence of exogenous resources. The secreted polysaccharides concentration was higher, peaking at 116.47 mg/L at 24 h and then dropping to 105.57 mg/L at 48 h. The reducing power followed the same pattern as the polysaccharides, peaking at 0.179 absorbance at 24 h and then falling to 0.140 at 48 h. In contrast, AuNPs were produced by reducing Au^3+^ to Au^0^. The current study [50] also confirms that mannose released by the yeast *Pichia pastoris* promotes the synthesis of AuNPs. As such, we suggested that the polysaccharides provided most of the reducing power in the reaction solution, and an increase in polysaccharide concentration was accompanied by significant AuNPs formation (the maximum absorption peaks of AuNPs are shown in Fig. 1). The concentration of secreted proteins in the experiment increased progressively, although the percentage proportion remained low, especially in the first 24 h. As the synthesis process of AuNPs proceeded, the concentration of proteins did not change much, only 13.49 mg/L, and these released proteins were thought to play a lesser role in the reduction process [42]. Furthermore, the maximum absorption peaks of AuNPs at 4 h, 12 h, 24 h, 36 h, and 48 h corresponded to wavelengths of 554 nm, 549 nm, 547 nm, 545 nm, and 544 nm (absorbance data of AuNPs from the maximum absorption peak of Fig. 1), with a slight blue shift in absorption wavelength. It suggests that the particle size distribution of AuNPs was more scattered [50], and that –NH_2_ groups or cysteine residues in proteins were involved in changing AuNPs morphology [51]. Thus, it could be hypothesized that XY3-released polysaccharides was a critical component that aided in AuNPs synthesis, while the secreted protein altered the AuNPs morphology and promoted AuNPs dispersion in solution.

### 3.3 Fungus XY3 mediated AuNPs synthesis under nystatin inhibition

**Fig. 3:**
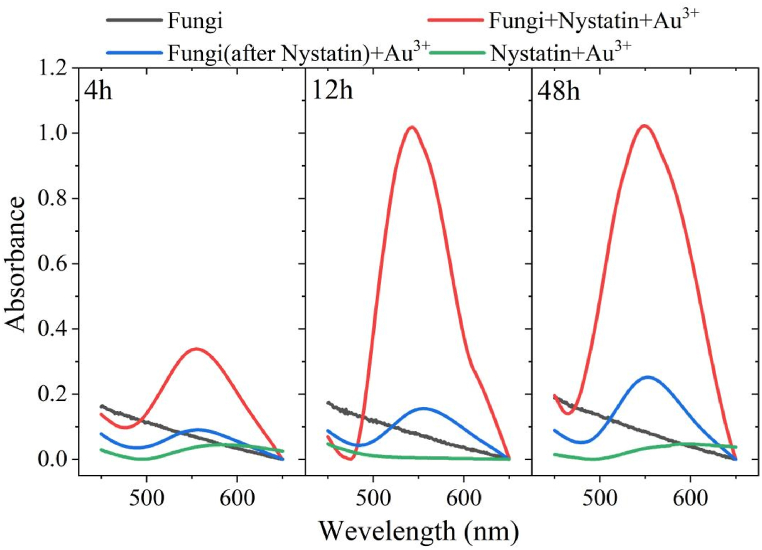
Influence of nystatin on the yield of AuNPs synthesized by the fungus XY3 under different treatments.

Nystatin is an antifungal antibiotic. The active site of nystatin is the ergosterol on the fungal cell membrane, which can selectively bind to it to form a permeable pore and enhance the permeability of the cell membrane, effectively suppressing the fungus's activities and causing the cytoplasm to leak out and die [52]. Two control tests, one with only fungus XY3 and the other with nystatin and Au^3+^, confirmed that there is no absorption peak at 540–560 nm and that it did not interfere with AuNPs determination (seen in Fig. 3). After 48 h treatment with nystatin, no growth of fungus XY3 was observed on solid medium, approving that nystatin had completely suppressed fungus XY3. The fungus XY3 treated with nystatin was mixed with Au^3+^, and AuNPs were detected at 4 h, 12 h, and 48 h with absorbances of 0.090, 0.156, and 0.251, respectively. It suggests that the residual cell wall, cell membrane, and other structures of the nystatin-treated fungus XY3 still have enough reducing power to enable AuNPs synthesis. Xie et al. [53] validated that protein-like biomolecules on the fungal cell wall involved in AuNPs production. When fungus XY3, nystatin, and Au^3+^ were added simultaneously, AuNPs were detected at 4 h, 12 h, and 48 h with absorbances of 0.337, 1.017, and 1.021, respectively. The yield of AuNPs was significantly higher than that of the test group that was first treated with nystatin. Because the inhibition of fungal growth by nystatin was not instantaneous and required time, the fungus XY3 continued to mediate AuNPs synthesis throughout the first 12 h. Furthermore, nystatin promoted the outflow of fungus XY3 intracellular materials at a faster pace, resulting in a larger AuNPs concentration. After the nystatin had entirely suppressed the fungus XY3, the content of AuNPs ceased to increase on the 12th h. Thus, this work demonstrated that fungus XY3 mediated AuNPs synthesis was primarily dependent on Au^3+^ stimulated endogenous metabolic activities, while small amounts of AuNPs were formed by surface structures such as cell walls and cell membranes.

### 3.4 Effects of XY3 surface structure on AuNPs synthesis

**Fig. 4:**
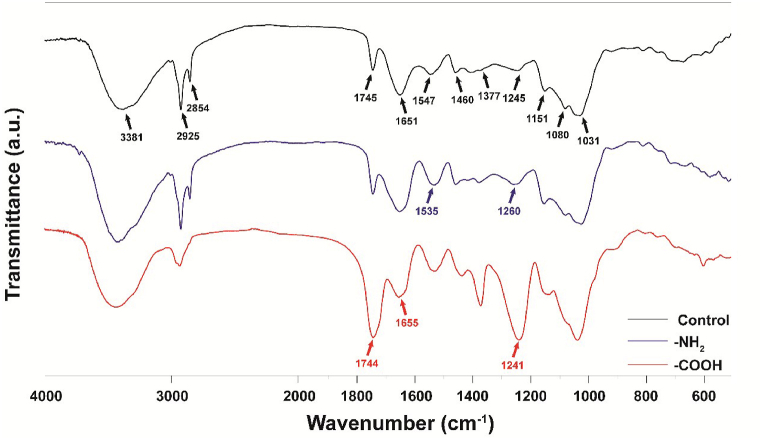
The FTIR spectra obtained before and after extracellular biomolecular modifications of fungus XY3.

**Fig. 5:**
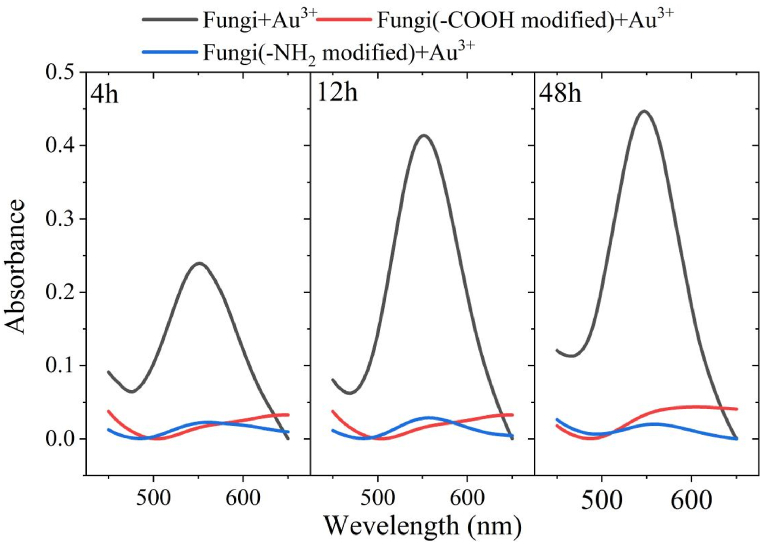
Effect of –COOH and –NH_2_ modifications on the yield of AuNPs synthesized by fungal XY3.

As shown in Fig. 4, Fig. 5, group modification was used to investigate the effect of fungal surface structure on AuNPs synthesis. The modification of functional groups on the surface of fungal XY3 was analyzed using FTIR. Fig. 4 indicated unmodified fungal surface structure. The unmodified fungal surface groups include: 1) –OH and –NH_2_ groups indicated by the larger absorption peak at 3381 cm^−1^; 2) expansion vibration of –CH indicated at 2925 cm^−1^; 3) stretching vibration of –CH at 2854 cm^−1^, while C=O stretching vibration of –COOH and –COO groups at 1745 cm^−1^; 4) C=O stretching vibration of amide I at 1651 cm^−1^, while N–H bending and C–N stretching vibration of amide II at 1547 cm^−1^; 5) –CH_3_ stretching vibration of acetyl group at 1460 cm^−1^; 6) –NH_2_ or sulfonyl group at 1377 cm^−1^; 6) –NH_2_ group or –CO group of –COOH at 1245 cm^−1^; 7) –CN group at 1151 cm^−1^; 8) –CO or –CN groups at 1080 cm^−1^, 1030 cm^−1^ [54,55]. It suggests that surface structure of fungus XY3 had conventional carbohydrate and protein biomolecules [56]. The more reactive hydrogen atom on –NH_2_ was replaced by –CH_3_, making the N atom more stable. The adsorption peak at 1547 cm^−1^ was shifted to 1535 cm^−1^ in FTIR spectrum, suggesting that the –NH_2_ group was impacted. The identical result of shift from 1245 cm^−1^ to 1260 cm^−1^ also indicated that –NH_2_ group was modified. The esterification reaction by adding ethanol converted –COOH to –COOCH_2_CH_3_, and the vibrations at 1744 cm^−1^ and 1655 cm^−1^ were enhanced and weakened, respectively. Compared to those before the modification, it indicated that the represented C=O and –CO were disrupted and the carboxyl group was converted to the ester group. The foregoing findings showed that modifying the –NH_2_ and –COOH groups on the surface of fungus XY3 was effective.

Fig. 5 shows a more detailed study of the AuNPs synthesis results. It suggests that AuNPs synthesis could be successfully mediated by unmodified fungus XY3. In tests of modified –COOH and –NH_2_ groups, no AuNPs was synthesized. Firstly, it implies that other groups on surface of fungus XY3 could not mediate AuNPs synthesis, including traditional physical and chemical reactions. Furthermore, –COOH and –NH_2_ were present in proteins, peptides and amino acids, which play an important role in maintaining the protein structure and activity. The denaturation or absence of a group would affect the protein's role in biometabolism [57]. Thus, it is suggested that the presence of reductase on the surface of fungus XY3 could promote AuNPs production by combining –COOH and –NH_2_. Above-mentioned reductase was also confirmed by Bansal et al. [58]. The –COO– group of reductase could electrostatically trap Au^3+^ and then reduce it to AuNPs [42]. As a result, surface structure-mediated AuNPs synthesis by fungus XY3 was a reductase-mediated biological reaction rather than a conventional chemical reduction reaction.

### 3.5 Process and mechanism of AuNPs synthesis mediated by fungus XY3

**Fig. 6:**
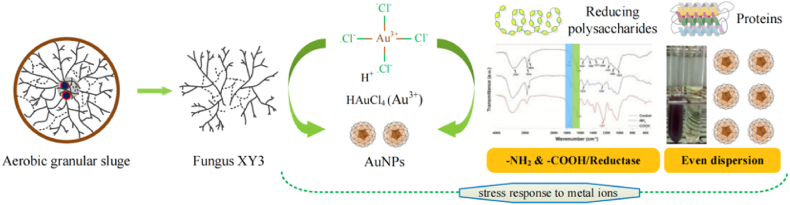
Under the stimulation of Au^3+^, the mechanism of AuNPs synthesis mediated by fungus XY3.

According to the above findings, the XY3-assisted AuNPs synthesis belonged to biochemical processes, which are depicted in Fig. 6. At present, according to the general strategy outlined for microbially driven nanoparticle synthesis, metal ions are trapped in microbial cells or on microbial surfaces in the presence of enzymes [59]. The transfer of electrons from NADH to an electron carrier (NADH-dependent reductase) is the initial step in the reduction process, followed by electron transfer to Au^3+^ and reduction to Au^0^ or AuNPs [60]. In this work, fungus XY3 mediated AuNPs synthesis was dependent on the stress response simulated by Au^3+^. Reducing polysaccharides were the key biomolecules for AuNPs synthesis, while extracellular proteins were the crucial biomolecules to improve nanoparticle even dispersion in solution. The presence of reductase in the fungus XY3 surface structure was comparable to the generic strategy described above, but only a small amount of AuNPs synthesis was mediated by it.

## 4 Conclusion

From fungal-type aerobic granular sludge, a fungal strain capable of efficiently reducing Au^3+^ and synthesizing AuNPs was screened and compared to *Candida rugopelliculosa* (NG063475.1) with 99% similarity, which named as XY3. During 48 h of incubation with 1 mM HAuCl_4_·4H_2_O, the concentration of AuNPs gradually increased while Au^3+^ was reduced. The nystatin inhibition assay demonstrated that the production of AuNPs by XY3 was a biochemical reaction. Subsequently, analysis of the changes in reducing power, polysaccharides and AuNPs indicated that the reducing polysaccharides secreted by XY3 were the main biomolecules for reducing Au^3+^ to Au^0^. According to the results of fungal cell surface moiety modification, it was demonstrated that extracellular enzymes were involved in the synthesis of AuNPs and promoted the uniform dispersion of AuNPs in the liquid phase.

## CRediT authorship contribution statement

**Xin Zhao:** Writing – original draft, Investigation. **Ning Hou:** Writing – review & editing, Supervision, Resources, Conceptualization. **Chunli Wan:** Methodology, Conceptualization. **Lei Zhang:** Writing – review & editing, Conceptualization. **Xiang Liu:** Writing – review & editing, Supervision, Resources, Conceptualization.

## Declaration of competing interest

The authors declare the following financial interests/personal relationships which may be considered as potential competing interests: Lei Zhang reports financial support was provided by 10.13039/501100001793Queensland University of Technology. Xin Zhao and Ning Hou reports financial support was provided by 10.13039/501100008534Northeast Agricultural University. Chunli Wan reports financial support was provided by 10.13039/501100003347Fudan University. Xiang Liu reports financial support was provided by 10.13039/501100003347Fudan University. If there are other authors, they declare that they have no known competing financial interests or personal relationships that could have appeared to influence the work reported in this paper.
