# Supplementary material for: Gold nanoparticles synthesis mediated by fungus isolated from aerobic granular sludge: Process and mechanisms
Source: Heliyon. 2024 Mar 16;10(6):e28281. doi: 10.1016/j.heliyon.2024.e28281 (PMC10966691; doi:10.1016/j.heliyon.2024.e28281)
Supplement: Multimedia component 1 [file mmc1.docx]

**Gold nanoparticles synthesis mediated by fungus isolated from aerobic granular sludge: Process and mechanisms**

Xin Zhao^a^, Ning Hou^a^, Chunli Wan^b^, Lei Zhang^c,*^, Xiang Liu^b,^^*^

^a^ College of Resources and Environment, Northeast Agricultural University, Harbin, Heilongjiang, 150030, China.

^b^ Department of Environmental Science and Engineering, Fudan University, Shanghai 200438, China.

^c^ School of Civil and Environmental Engineering, Queensland University of Technology, Brisbane, 4001, Australia

*Corresponding authors: Xiang Liu (liuxiang@fudan.edu.cn) and Lei Zhang (ZhangL53@qut.edu.au)


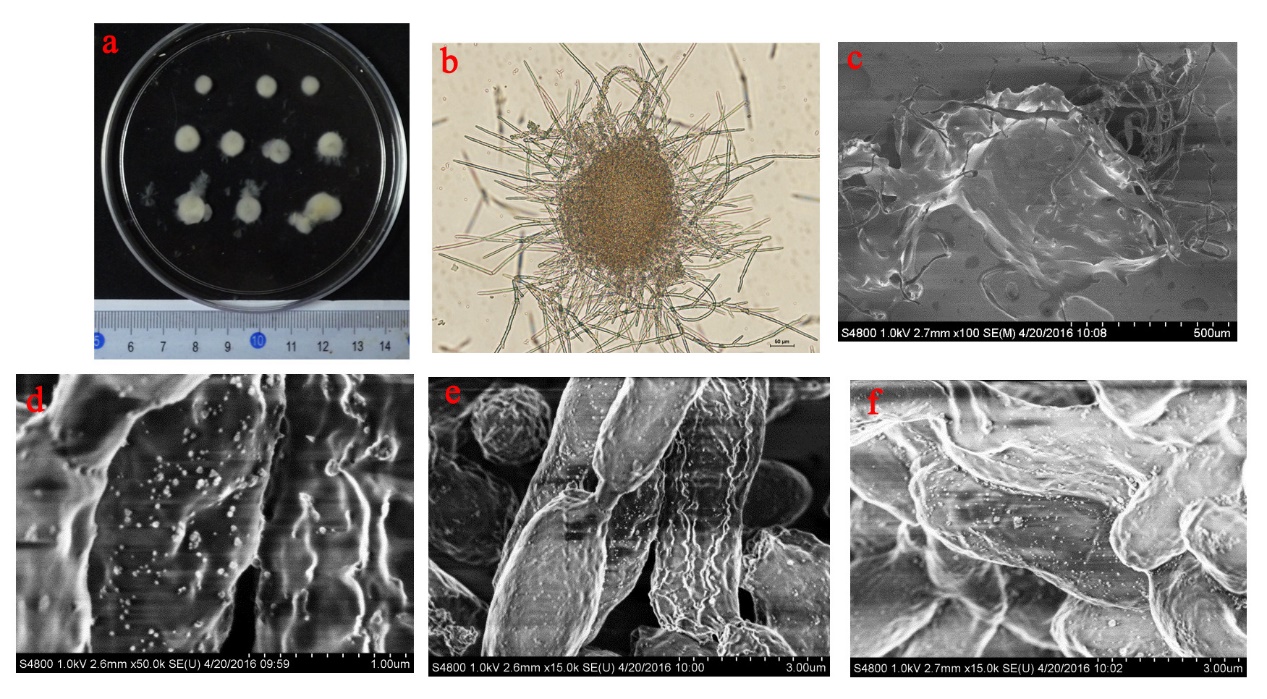


Fig. S1 Morphological characteristics of aerobic granules, fungus XY3, and fungus-mediated AuNPs. a: digital picture of aerobic granules; b: microscope photo of fungus XY3; c: SEM picture of fungus XY3; d-f: SEM pictures of fungus-mediated AuNPs.


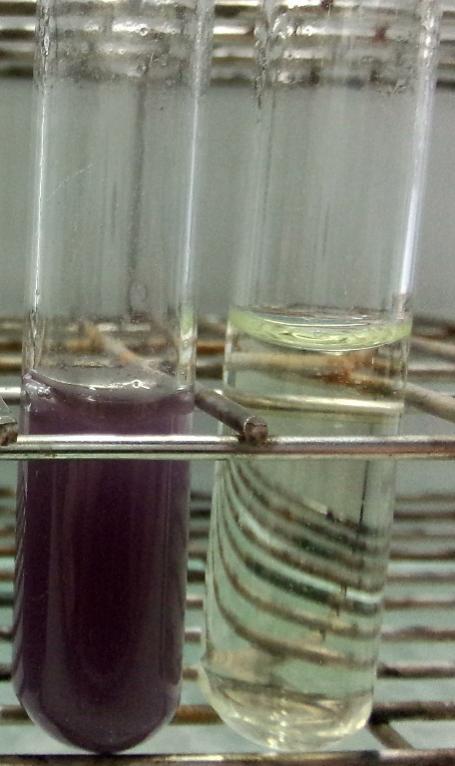


Fig. S2 Practical effect of fungus-mediated Au^3+^ reduction and AuNPs synthesis. Left: solution contained AuNPs; Right: HClAu_4_ solution.
